# Video Grading of Pancreatic Anastomoses During Robotic Pancreatoduodenectomy to Assess Both Learning Curve and the Risk of Pancreatic Fistula: A Post Hoc Analysis of the LAELAPS-3 Training Program

**DOI:** 10.1097/SLA.0000000000005796

**Published:** 2023-01-20

**Authors:** Bram L.J. van den Broek, Maurice J.W. Zwart, Bert A. Bonsing, Olivier R. Busch, Jacob L. van Dam, Ignace H.J.T. de Hingh, Melissa E. Hogg, Misha D. Luyer, J.Sven D. Mieog, Luna A. Stibbe, Kosei Takagi, T. C. Khe Tran, Roeland F. de Wilde, Herbert J. Zeh, Amer H. Zureikat, Bas Groot Koerkamp, Marc G. Besselink

**Affiliations:** * Department of Surgery, Erasmus University Medical Center, Rotterdam, Netherlands; † Department of Surgery, Amsterdam UMC, University of Amsterdam, Amsterdam, Netherlands; ‡ Cancer Center Amsterdam, Amsterdam, Netherlands; § Department of Surgery, Leiden University Medical Center, Leiden, Netherlands; ∥ Department of Surgery, Catharina Medical Center, Eindhoven, Netherlands; ¶ Department of Surgery, NorthShore University HealthSystem, Evanston, IL; # Department of Surgery, University of Texas Southwestern Medical Center, Dallas, TX; ** Department of Surgery, Division of GI Surgical Oncology, University of Pittsburgh Medical Center, Pittsburgh, PA

**Keywords:** OSATS, pancreaticojejunostomy, robotic pancreatoduodenectomy

## Abstract

**Objective:**

To assess the learning curve of pancreaticojejunostomy during robotic pancreatoduodenectomy (RPD) and to predict the risk of postoperative pancreatic fistula (POPF) by using the objective structured assessment of technical skills (OSATS), taking the fistula risk into account.

**Background:**

RPD is a challenging procedure that requires extensive training and confirmation of adequate surgical performance. Video grading, modified for RPD, of the pancreatic anastomosis could assess the learning curve of RPD and predict the risk of POPF.

**Methods:**

Post hoc assessment of patients prospectively included in 4 Dutch centers in a nationwide LAELAPS-3 training program for RPD. Video grading of the pancreaticojejunostomy was performed by 2 graders using OSATS (attainable score: 12–60). The main outcomes were the combined OSATS of the 2 graders and POPF (grade B/C). Cumulative sum analyzed a turning point in the learning curve for surgical skill. Logistic regression determined the cutoff for OSATS. Patients were categorized for POPF risk (ie, low, intermediate, and high) based on the updated alternative fistula risk scores.

**Results:**

Videos from 153 pancreatic anastomoses were included. Median OSATS score was 48 (interquartile range: 41–52) points and with a turning point at 33 procedures. POPF occurred in 39 patients (25.5%). An OSATS score below 49, present in 77 patients (50.3%), was associated with an increased risk of POPF (odds ratio: 4.01, *P*=0.004). The POPF rate was 43.6% with OSATS < 49 versus 15.8% with OSATS ≥49. The updated alternative fistula risk scores category “soft pancreatic texture” was the second strongest prognostic factor of POPF (odds ratio: 3.37, *P*=0.040). Median cumulative surgical experience was 17 years (interquartile range: 8–21).

**Conclusions:**

Video grading of the pancreatic anastomosis in RPD using OSATS identified a learning curve and a reduced risk of POPF in case of better surgical performance. Video grading may provide a valid method to surgical training, quality control, and improvement.

Postoperative pancreatic fistula (POPF) occurs in up to 26% of patients after pancreatoduodenectomy (PD).^1^ Various scores have been developed for predicting POPF, including the fistula risk score,^2^ the updated alternative fistula risk score (uaFRS),^3,4^ and the clinical risk score for pancreatic fistula.^2,5^ However, these prediction models do not take technical skills into account. It is known that both the complexity of the procedure, and the proficiency necessary to successfully perform the procedure influence outcome.^6,7^ Few studies have directly assessed the association between technical surgical skills and clinical outcomes.^8^ In recent years, PD is increasingly performed via the minimally invasive approach (laparoscopic or robot assisted), which enables high-quality video recording of the procedure and could provide an opportunity to assess technical skills and thereby predict POPF.

For laparoscopic surgery, Birkmeyer et al^9^ investigated the objective structured assessment of technical skills (OSATS) and demonstrated fewer complications when patients were operated by surgeons with the highest skill rating compared with the lowest skill rating. The University of Pittsburgh Medical Center (UPMC) applied the Birkmeyer method for robotic pancreatoduodenectomy (RPD) using a modified OSATS score and concluded that proficiency in robotic pancreaticojejunostomy correlated with the incidence of POPF.^10^ This method could quantify surgical skill performance in robotic surgery, ultimately validating surgeon skills within teaching programs. However, multicenter studies on external validation of the modified OSATS score in RPD are lacking. Furthermore, a validated performance cutoff of the OSATS score for the risk of POPF is lacking.

The objective of this study was to assess surgical skills and prediction of POPF by video grading of the pancreaticojejunostomy anastomosis during RPD using the OSATS, and to find a safe OSATS performance cutoff value and validating the modified OSATS score for RPD.

## METHODS

### Study Design

This is a post hoc assessment of prospectively collected data from the nationwide LAELAPS-3 training program for RPD (NTR8073). This study included all consecutive RPD data from 4 centers in the Netherlands (Erasmus Medical Center Rotterdam, Amsterdam UMC, Leiden University Medical Center, Catharina Hospital Eindhoven) between January 2017 and June 2020. The study was approved by the institutional review boards of the participating centers.

### Patients, Surgeons, and Variables

Patients who underwent RPD for all indications were eligible. Patients were excluded when no video was available for the complete pancreaticojejunostomy using the modified Blumgart method, which includes 3 posterior mattrass sutures (of which the middle one surrounds the pancreatic duct), a minimum of 5 duct-to-mucosa sutures with an intraductal stent, and 3 anterior buttress sutures.^11^ This was defined by the following steps: (1) setting up the bowel, (2) posterior mattress, (3) jejunal enterotomy, (4) duct-to-mucosa sutures, (5) anterior mattress. In addition, each patient’s updated alternative FRS (uaFRS) score was calculated using the following variables: sex, body mass index (BMI), duct size, and pancreas texture.^4^ The uaFRS categorizes patients at low risk (<5%), intermediate risk (5%–20%), and high risk (>20%) of POPF. The American Society of Anesthesiologist Physical Status Classification System was used for the baseline physical status of the patient.^12^ All patients received an internal pancreatic duct stent.^11^ In 74% of patients, 1 or 2 postoperative drains were placed, for which removal was according to the PORSCH trial algorithm,^13^ or when not in an including center or a control center according to Ven Fong et al^14^ (if POD 1 drain amylase level is <600 U/L, drains were removed on POD 1.). In 1 center, surgeons performed a minimum of 5 pancreaticojejunostomies on artificial organs before initiating the RPD program.^15^ Four surgeons performed pancreaticojejunostomies from the robotic console. However, in 1 center 2 additional surgeons performed only 1 pancreaticojejunostomy on artificial organs before performing their first RPD. Therefore, we included these cases in learning curve analysis as number 1 from these surgeons. Octreotide was administered in 70% of patients, tissue glue was used in 1 center, and in all centers the teres ligament was placed between the gastroduodenal artery stump and the pancreatic anastomosis.

### Grading

The OSATS score, which is widely used in assessing the skill of surgical trainees,^16^ predicted complications after bariatric procedures, and RPD in a modified version.^9,10^ The modified OSATS is comprised of 6 variables: gentleness, time and motion, instrument handling, flow of operation, tissue exposure, and the summary score (Table 1). For each variable, a score from 1 (deficient/traumatic) to 5 (master/flawless) could be awarded. The total OSATS score from each grader was calculated by adding up the 6 variables from their OSATS score. Two graders independently graded videos of the pancreaticojejunostomy. The first grader (M.J.W.Z.) was trained in OSATS grading in the UPMC for 10 days, and during that period and beyond, was trained on how to perform an optimal pancreaticojejunostomy by Melissa Hogg, Amer Zureikat, and Herbert Zeh. The second grader (B.A.B.) was trained by the first grader and studied both the Birkmeyer et al^9^ and Hogg et al^10^ OSATS publications, of which the former contains a detailed crash-course video on how to perform OSATS grading including examples of low to high performance.^9^ The intragrader reliability of the second grader was assessed after 20 pancreaticojejunostomies. The grader was also present at an RPD every week, during a period of 6 months. Both graders were blinded for patient outcomes, participating center, and the operating surgeon by anonymizing the videos with a random 4-digit code generated in SPSS. The combined OSATS score of the 2 graders was used in the calculations, attainable scores were 12 to 60. Intergrader correlation coefficient through Spearman Rho was interpreted as follows: +1 indicates a perfect association, 0 indicates no association between ranks, and −1 indicates a perfect negative association. The closer is to zero, the weaker the association between the ranks.

**TABLE 1:** Grading Definition OSATS

Rating and interpretation	
1	Deficient/traumatic
2	Lacking/lacks finesse
3	Average
4	Skilled
5	Master/flawless
Grading aspects and elucidation	
Gentleness	Gentle tissue handling that does not result in injury
Time and motion	Economy of motion, maximum efficiency
Instrument handling	Fluid use of instruments without awkwardness
Flow of operation	Smooth transitions from one part of the operation to another
Tissue exposure	Retraction that allows for good visualization and proper tissue alignment
Overall technical skill	Overall assessment of technical skill

Modified OSATS grading as reported by Hogg et al.^9,10^

### Outcomes

The primary outcome was the proportion of POPF (ISGPS grade B/C) in each quartile of the combined OSATS score.^17^ Secondary outcomes were the learning curve analysis and the intergrader variability of the OSATS score. Data were collected up to 90 days postoperatively.

### Statistical Analysis

Data were analyzed using IBM SPSS statistics for Windows version 27 (IBM Corp, Armonk, NY). Normally distributed continuous data are presented as mean and SD. Non-normally distributed continuous data are presented as median and interquartile range (IQR) or 95% CI. Categorical (binary, nominal, and ordinal) data are presented as frequencies and percentages. Likert-scale ordinal data were also presented in means and standard deviations, as this allows more insight into the effect size.^18^ Outcomes are compared and assessed for significance (2-tailed *P* value of <0.05) with Student *t* test for normally distributed data, χ^2^ for frequencies in 1 or more categories, and Mann-Whitney *U* test for non-normally distributed data. The OSATS score was categorized into 4 groups by quartiles (quartile 1, quartile 2, quartile 3, and quartile 4). Linear regression was used to test the association between the elements of the uaFRS and OSATS, case number, and mitigating strategies, as means to identify any back-door associations caused by confirmation bias, such as better performance in case of wider pancreatic ducts (survival bias) or lower grades in case of a soft pancreas (confirmation bias). Logistic regression was used to test the predictability from the OSATS. For the POPF rate per quartile and the intergrader correlation coefficient analysis, a Spearman rho was expressed. Learning curve analyses were performed to investigate the association of the OSATS score and the consecutive case number of each hospital. Cumulative sum (CUSUM) analysis was used to determine the learning curve turning point for the OSATS score. The turning point was defined as the moment when the learning started to decrease in slope angle. The performance cutoff analysis was performed by logistic regression (including uaFRS, mitigating strategies, and consecutive patients per surgeon) to identify an OSATS score to be reached to minimize the risk of POPF. We performed a sensitivity analysis excluding patients with neoadjuvant chemo(radio)therapy.

## RESULTS

**FIGURE 1:**
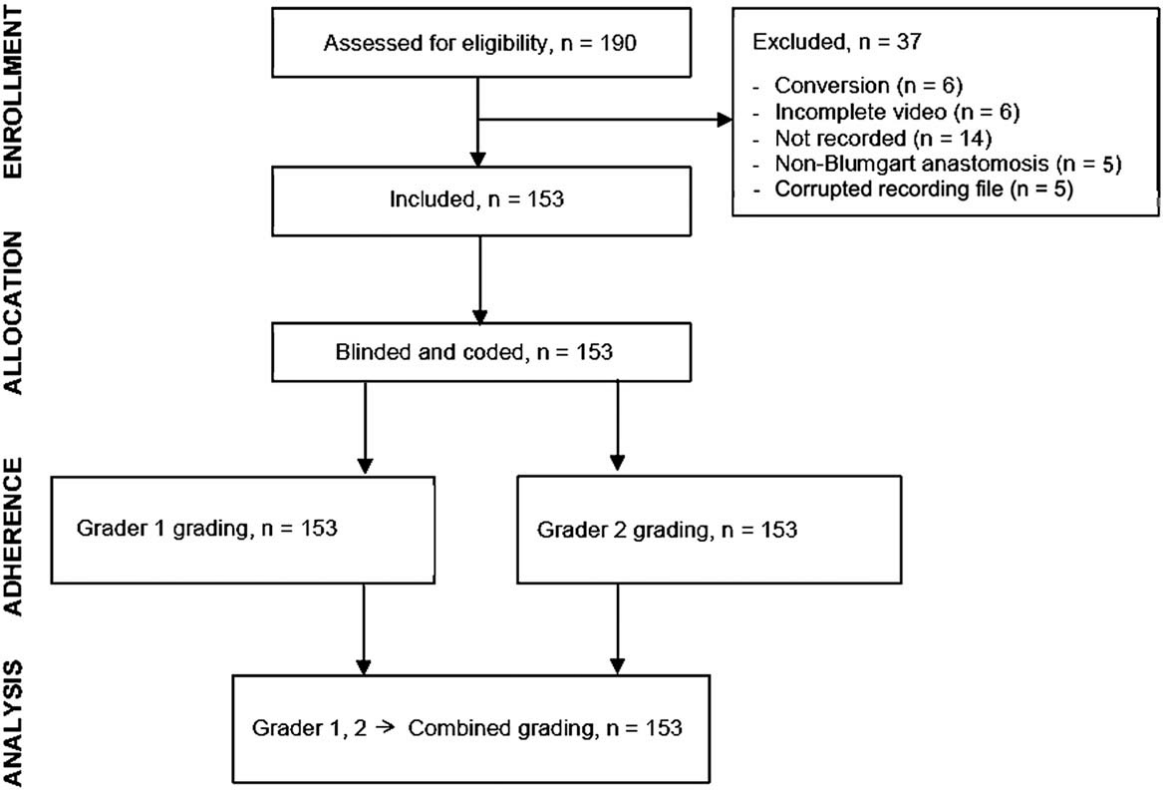
Flowchart of enrollment.

Overall, 190 patients underwent RPD, of whom 153 patients could be included. Patients were excluded for the following reasons: conversion to open surgery before pancreaticojejunostomy creation (n=6), incomplete video (n=6), pancreaticojejunostomies not performed with modified Blumgart technique (n=5), not recorded (n=14), and corrupted recording file (n=6) (see Figure 1 for a detailed overview of enrollment). POPF occurred in 41 patients (26.8%): grade B=34 (22.2%) and grade C=7 (4.6%). The 153 pancreaticojejunostomy anastomoses were performed by 6 surgeons with a median cumulative experience of 17 years [IQR: 8–21] at their first anastomosis. Three surgeons had experience with laparoscopic PD.

### Baseline Characteristics

See Table 2 for details on patient characteristics. The median patient BMI was 25 kg/m^2^ [IQR: 23–28]. On the basis of the uaFRS criteria, 13/153 (8.5%) patients were at low risk, 43/153 (28.1%) at intermediate risk, and 97/153 (63.4%) and high risk of POPF. The actual rate of POPF in the low-risk group was 7.7% (n=1/13), 11.6% (n=5/43) in the intermediate-risk group, and 34.0% (n=33/97) in the high-risk group.

**TABLE 2:** Baseline Characteristics of 153 Patients After Robotic Pancreatoduodenectomy

Characteristic	n=153
Age, years, median [IQR]	67 [60–73]
BMI, kg/m^2^, median [IQR]	24.9 [22.7–27.8]
Male, n (%)	85 (55.6)
Patients with intermediate to high risk^*^, n (%)	72 (47.1)
Indication, n (%)	
PDAC	48 (31.4)
Ampullary/duodenal cancer	40 (26.1)
Distal cholangiocarcinoma	17 (11.1)
IPMN	20 (13.1)
Pancreatic duct diameter, mm [IQR]	3 [2–5]
Pancreatic texture, n (%)	
Soft/normal	100 (65.4)
Hard/fibrotic	53 (34.6)
ASA physical status, n (%)	
1/2	11/93 (69.4)
3/4	45/1 (30.6)
Previous abdominal surgery, n (%)	61 (39.9)
Neoadjuvant chemo(radio)therapy, n (%)	15 (9.8)

* Based on uaFRS defined by Mungroop et al.^4^

ASA indicates American Society of Anesthesiologist Physical Status Classification System; IPMN, intraductal papillary mucinous neoplasm; PDAC, pancreatic ductal adenocarcinoma.

### OSATS

The awarded OSATS scores of both graders for the pancreatic anastomoses did not differ significantly, median 24 [IQR: 21–27] versus 24 [IQR: 20–26] points (*P*=0.322). Intergrader reliability was fair to moderate (95% CI, 0.52–0.75), intragrader reliability of the second grader demonstrated significant correlation, *P*=0.017 (average discrepancy +1.06=4.2%, intragrader reliability: 0.541, 95% CI, 0.115–0.799). The median combined OSATS score of both graders was 48 [IQR: 41–52] and OSATS scores ranged from 28 to 59 points. The combined OSATS scores did not demonstrate a correlation with the components of the uaFRS: duct size (*P*=0.626), pancreas texture (*P*=0.454), age (*P*=0.648), BMI (*P*=0.274), nor sex (*P*=0.106). For more details on the correlation of duct size versus OSATS, see Supplementary Digital Content 3, http://links.lww.com/SLA/E419.

### Baseline OSATS per Surgeon, Center, and Experience

Median OSATS scores per surgeon ranged from 41 to 50, without significant differences between surgeons (*P*=0.357) and centers (*P*=0.273). Furthermore, there was no significant differences in POPF rates per surgeon (*P*=0.097) and per center (*P*=0.120). Surgeons with experience with in laparoscopic PD did not differ in OSATS scores (*P*=0.860).

### OSATS and POPF

The combined OSATS scores were categorized in quartiles. The median OSATS scores in the quartiles were 38, 46, 51, and 55 points, and a corresponding POPF B/C rate of 26.3%, 43.7%, 15.8%, and 15.8%, respectively, Spearman rho: −0.160, *P*=0.015. See the figure in Supplementary Digital Content 1 (http://links.lww.com/SLA/E417) for more details. For grader 1, the decrease in POPF per OSATS quartile was significant (Spearman rho: −0.160, *P*=0.015). For grader 2, this decrease was not significant (Spearman rho: −0.113, *P*=0.167). Between 4 groups, age, BMI, American Society of Anesthesiologist, previous abdominal surgery, proportion of PDAC, and uaFRS did not differ significantly. With the increase of quartiles, there were significantly more patients who received neoadjuvant chemo(radio)therapy, and RPDs were performed later in the experience (median 15th, 31st, 62nd, 62nd, respectively, *P*<0.001) (Table 3). The median duration of a pancreaticojejunostomy in the quartiles was 45, 54, 45, and 44 minutes, respectively, for quartiles 1, 2, 3, and 4, *P*<0.001. A pancreaticojejunostomy in the highest category (quartile 4) was 28% faster than in the lowest quartile (39 vs. 54 minutes, *P*<0.001).

**TABLE 3:** Characteristics of Patient Surgery, According to Rating of Surgical Skill

	OSATS					
Baseline characteristic	Total (n=153)	Quartile 1 (n=38)	Quartile 2 (n=39)	Quartile 3 (n=38)	Quartile 4 (n=38)	*P*
Age, years, median [IQR]	67 [61–74]	68 [61–73]	66 [59–74]	70 [60–73]	67 (59–74)	0.847
BMI, kg/m^2^, median [IQR]	24 [23–28]	25 [23–29]	25 [22–26]	25 [23–28]	25 (23–28)	0.181
Male, n (%)	85 (55.6)	26 (58.4)	18 (48.7)	24 (63.2)	17 (44.7)	0.099
ASA physical status ≥ 3, n (%)	47 (30.7)	10 (26.3)	12 (30.8)	12 (33.3)	13 (34.2)	0.783
Previous abdominal surgery, n (%)	61 (39.9)	15 (39.5)	20 (51.3)	15 (39.5)	11 (28.9)	0.216
Neoadjuvant chemo(radio)therapy, n (%)	15 (9.8)	3 (7.9)	1 (2.6)	2 (5.3)	9 (23.7)	0.009
PDAC, n (%)	48 (28.4)	11 (28.9)	9 (23.1)	12 (31.6)	16 (42.1)	0.336
POPF, n (%)	35 (22.9)	10 (26.3)	17 (43.7)	6 (15.8)	6 (15.8)	0.033
uaFRS, mean (95% CI)	0.31 (0.28–0.34)	0.32 (0.25–0.40)	0.34 (0.28–0.41)	—	0.29 (0.25–0.33)	0.390
Surgical characteristic						
Operative time, min, median [IQR]	412 [380–481]	420 [387–513]	405 [382–520]	416 [364–458]	406 [365–469]	0.337
PJ time, min, median [IQR]	45 [39–54]	54 [47–64]	45 [40–57]	44 [38–46]	39 [35–45]	<0.001
Estimated blood loss, mL, median [IQR]	200 [100–450]	300 [125–475]	200 [100–500]	200 [100–400	200 [100–450]	0.402
Median number of operations by surgeon [IQR]	34 [14–66]	15 [6–37]	31 [14–52]	62 [36–83]	62 [36–83]	<0.001
Grading (combined OSATS score)						
Total OSATS score, median [IQR]	48 [41–52]	38 [33–40]	46 [45–47]	50 [50–52]	54 [53–56]	<0.001
Gentleness mean (95% CI)	7.5 (7.2–7.7)	6.1 (5.6–6.6)	7.5 (7.0–7.9)	7.9 (7.5–8.2)	8.1 (7.9–8.4)	<0.001
Time and motion mean (95% CI)	8.0 (7.8–8.3)	6.2 (5.9–6.5)	7.7 (7.5–8.1)	8.7 (8.4–9.0)	9.1 (8.9–9.3)	<0.001
Instrument handling mean (95% CI)	8.1 (7.8–8.3)	6.5 (6.1–6.8)	7.8 (7.5–8.1)	8.5 (8.2–8.8)	9.0 (8.8–9.1)	<0.001
Flow of operation mean (95% CI)	7.9 (7.6–8.1)	6.3 (5.8–6.8)	7.6 (7.2–8.0)	8.5 (8.1–8.9)	8.8 (8.5–9.0)	<0.001
Tissue exposure mean (95% CI)	8.1 (7.8–8.4)	6.1 (5.6–6.6)	8.0 (7.5–8.5)	8.8 (8.4–9.1)	9.2 (9.0–9.4)	<0.001
Summary score mean (95% CI)	7.4 (7.2–7.6)	5.5 (5.2–5.8)	7.1 (6.9–7.3)	8.2 (8.0–8.3)	8.5 (8.4–8.7)	<0.001

ASA indicates American Society of Anesthesiologist Physical Status Classification System;^12^ mL, milliliters; PDAC, pancreatic ductal adenocarcinoma; PJ, pancreaticojejunostomy; POPF, Postoperative pancreatic fistula.

**TABLE 4:** Predictive Value of OSATS

	Univariable		Multivariable	
Characteristic	Odds ratio	*P*	Odds ratio	*P*
Consecutive RPDs increments	0.01^*^	0.611	1.01	0.243
OSATS quartiles	-0.15^*^	0.033	—	—
Quartile 1^†^	1.91	0.111	3.22	0.127
Quartile 2^†^	4.12	0.008	5.55	0.012
Quartile 3^†^	1.00	1.000	1.12	0.875
Quartile 4=Ref	—	—	—	—
uaFRS				
Age, year increments	1.00	0.874	1.00	0.706
BMI, kg/m^2^ increments	1.04	0.411	1.07	0.243
Sex (male)	1.33	0.446	1.28	0.605
Pancreas texture (soft)	5.49	<0.001	3.37	0.040
Duct size mm increments	0.83	0.046	0.96	0.286
Mitigating strategies				
Tissue glue	1.07	0.919	0.93	0.929
Teres ligament patch	1.70	0.359	0.53	0.240
Somatostatin analog	1.22	0.643	0.67	0.547
Postoperative drain	0.96	0.819	0.24	0.193
OSATS performance cutoff				
Cutoff 46	1.59	0.216	2.28	0.082
Cutoff 47	1.49	0.019	3.33	0.012
Cutoff 48	1.66	0.010	3.99	0.004
Cutoff 49	3.11	0.004	4.01	0.004
Cutoff 50	2.33	0.031	3.16	0.018
Cutoff 51	1.57	0.100	2.77	0.042
Cutoff 52	1.29	0.279	1.48	0.444

* Spearman rho.

† Values are relative to quartile 1.

On univariable analysis, this risk of POPF in the lowest OSATS quartiles was higher as compared with the highest OSATS quartiles, although not significant (OR: 1.91, 95% CI, 0.84–5.44, *P*=0.111). However, the second quartile had a significantly higher risk of POPF as compared with the highest OSATS quartile (OR: 4.12, 95% CI, 1.40–12.11, *P*=0.008). The third quartile had identical risk of POPF (OR: 1.00, 95% CI, 0.291–3.43, *P*=0.008). On multivariable analysis a higher risk for developing POPF was found for OSATS quartiles 1, quartile 2, and quartile 3 as compared with quartile 4: OR: 3.22, *P*=0.127; OR: 5.55, *P*=0.012, OR: 1.12, *P*=0.875, respectively. The soft pancreas texture remained as a strong prognostic factor of POPF (OR: 3.37, *P*=0.001). In a sensitivity analysis of the high risk uaFRS group (n=96) only, the OSATS score quartiles 1 to 4 corresponded to POPF B/C rates of 39.1%, 53.8%, 18.5%, and 25.0%, respectively, *P*=0.039. A multivariable analysis with only the variables that demonstrated *P<*0.100 on univariable analysis, revealed consistent results: quartile 2 OR: 3.88, *P*=0.027; soft pancreas texture OR: 3.72, *P*=0.021. A sensitivity analysis excluding patients who received neoadjuvant chemo(radio)therapy found no difference in the predictive value of the OSATS score (see Supplementary Digital Content 2, http://links.lww.com/SLA/E418 for more details).

The highest scores in patients without POPF, grade B POPF, and grade C POPF were 59, 56, and 52, respectively. The performance cutoff analysis showed a significant reduction in the risk of POPF for OSATS performance cutoffs 47 to 51, *P* < 0.050. The performance cutoff analysis showed that for an OSATS below 49 the risk of POPF was increased OR: 4.01 (95% CI, 1.44–6.71, *P*=0.004) (Table 4). The POPF of 43.6% in OSATS < 49 versus 15.8% in OSATS ≥49 resulted in a risk reduction of 27.8% (95% CI, 7.9%–59.6%). Multivariable performance cutoff analysis adjusted for uaFRS showed that for OSATS score below 49 the risk of POPF was increased, OR: 4.01 (95% CI, 1.53–10.22), *P*=0.004.

### OSATS and Learning Curve

**FIGURE 2:**
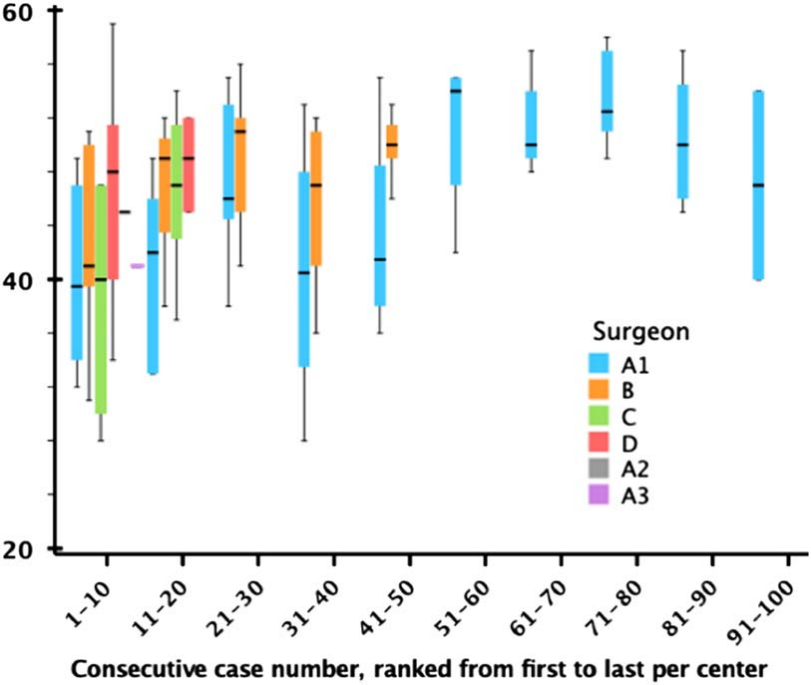
Combined OSATS score in consecutive robotic pancreaticojejunostomies. The *x*-axis indicates groups of 10 consecutive cases, color indicated per surgeon up to the inclusion number (Surgeons A1–3 were from the same center) ranked from first to last per center, and the *y*-axis indicates the combined OSATS score. The black line indicates the median OSATS scores with interquartile range box and range brackets. Min 12 to Max 60.

**FIGURE 3:**
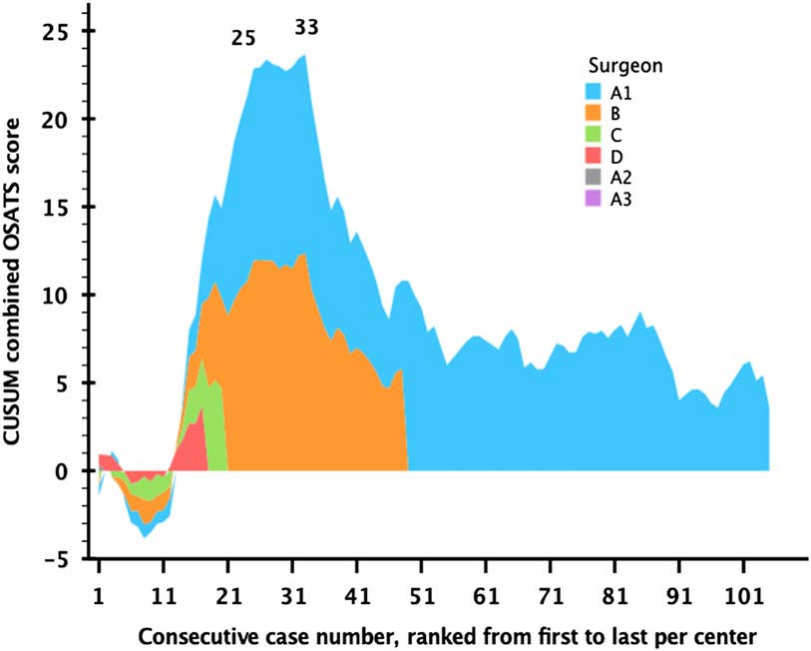
OSATS CUSUM analysis during robot pancreatoduodenectomy. The *x*-axis indicates consecutive cases of all centers, color indicated per surgeon up to the inclusion number (Surgeons A1–3 were from the same center). The *y*-axis indicates the CUSUM analysis for OSATS. The first label (n=25) indicates the first top turning point of the learning curve, where after, stabilization of the learning curve occurs. Hereafter, the second label (n=33) indicates the turning point where the learning curve follows a continuous downward slope.

Figure 2 demonstrated the OSATS score for each 10 consecutive RPDs per surgeon. CUSUM analysis of OSATS revealed an upward slope after 11 RPD procedures and a stabilization after 25 procedures, after which the learning reached a continuous downward slope. It also showed a turning point of the learning curve at 33 RPDs (Fig. 3). This indicates the end of the learning curve at 33 procedures. Of the different items in the OSATS score, gentleness in the highest quartile had the lowest mean score of 8.1 points suggesting that gentleness is the final variable to reach the mastery phase. The tissue exposure had the highest difference between the quartiles, with a mean of 6.1 points in the lowest quartile, and 9.2 points in the highest quartile.

## DISCUSSION

This multicenter study found that video grading of the pancreatic anastomosis during RPD using the OSATS score, reflecting surgical skills, identified a learning curve, could be used as a performance cutoff value to the risk of POPD and could predict the risk of POPF. The uaFRS risk score, reflecting pancreatic variables, remained the strongest predictor of POPF.

This is the first multicenter study to correlate surgical performance assessed through OSATS with the complication POPF. Birkmeyer applied the OSATS in 2013 for bariatric surgery, and in 2016 Hogg used it for the first time in pancreatic surgery.^9,10^ In a single-center study, Hogg et al^10^ used postlearning curve videos (all surgeons had performed at least 30 pancreaticojejunostomies to become eligible for inclusion), and could therefore not asses a learning curve effect. By including the initial 30 RPD procedures of 6 surgeons who all participated in the LELAPS-3 training program for RPD, we demonstrated how technical skills evolve in the learning curve. However, it should be noted that the learning curve in our cohort might be shorter than in routine clinical practice, as the surgeons in our cohort mostly performed 5 pancreaticojejunostomies on artificial organs before their first RPD procedure. Furthermore, it is critical to note that the cumulative experience before the first RPD was high (17 years). This is important to recognize especially for surgeons coming out of training as they start building their practice and program. Until now, no OSATS safety performance level existed for RPD. By adhering to the OSATS safety performance level (performance cutoff 49), a 28% reduction of POPF could be obtained. These results were generated by both graders blinded for performing surgeon, center, and patient outcomes. The results remained consistent after including a sensitivity analysis of the uaFRS.

It may be of interest to compare the present findings in 153 patients to the previous study from the UPMC group in (133 patients). The rate of POPF in the highest OSATS quartile (16%) in this study was somewhat higher than in the UPMC series (9%), although the uaFRS scores were comparable. In other anastomoses (eg, the gastrojejunostomy) anastomotic complications could be explained by technical factors rather than surgical performance alone.^19^ This suggests that technical factors while constructing the pancreaticojejunostomy, might also influence the occurrence of POPF.

Similar to the current study, the UPMC method for pancreaticojejunostomy grading demonstrated a significant correlation between OSATS and POPF. However, this was the case in only 1 of 2 graders.^10^ Difference in outcomes between graders could suggest the OSATS grading system demonstrates low intergrader reliability. In the current study, graders were not excluded to limit the chance for grader selection bias. Also in our study, only one of the graders was individually capable of predicting for POPF, with a moderate correlation between the 2 graders. Combined scores, as a way to limit intergrader differences, revealed a predictive score for POPF (Spearman rho: −0.160, *P*=0.015). This suggests that the OSATS could be unreliable for grading by a single grader, but becomes more valuable when multiple graders are involved. The latter is further supported by literature on other surgical procedures where grading methods also demonstrated a correlation between OSATS, and postoperative complications.^19–22^ For an overview of such studies, see Supplementary Digital Content 4, (http://links.lww.com/SLA/E420). In the future, artificial intelligence systems may be able to objectively determine the OSATS score.

Both the individual graders and the combined scores identified a learning curve during consecutive cases. After the CUSUM turning point (n=33), above-average scores were demonstrated. However, the mean OSATS scores further improved up to 60 procedures. This suggest that a surgeon is less likely to make a mistake in the pancreaticojejunostomy after 60 RPD procedures. Similarly, Zhang et al^23^ found a completed learning curve for pancreaticojejunostomy during RPD after 60 procedures. This further supports the value of OSATS in learning curve analysis.

The OSATS score could be helpful for new surgeons who are starting with robotic pancreatic surgery. Our learning curve could provide guidance for new surgeons to verify their performance. There is a standardized technique and “patient” simulation during LAELAPS-3 training, that is, 5 mm duct size and soft pancreas. Thus, the performance could be impacted by elements of the uaFRS, as smaller ducts are more difficult to perform, potentially resulting in a lower OSATS. However, we could not find a relationship between the duct size and the OSATS score.

Besides, it is possible to get a near perfect score on the OSATS yet still have a patient develop POPF, as shown by the highest score of 55/60 in de grade B POPF patients. This further conforms that there are other factors contributing to the development of POPF, such as the factors included in the uaFRS. It could then be hypothesized that mitigating strategies are essential in aiming to further reduce the impact of POPF caused by patient factors alone. These include internal stents, external stents, somatostatin use, biological glues, and placing a teres ligament patch.^24^

With this study, we validated the results of Hogg and colleagues We built on their work by correcting the OSATS score for the fistula risk score in the multivariable analysis. This implicated that the OSATS is an independent risk factor for clinically relevant POPF (grade B and grade C). Also, the study of Hogg and colleagues looked at all the POPF (including grade A, now known as “biochemical leak”), where we only included clinically relevant fistula. This increases the clinical impact of the OSATS score. Also, as mentioned before, the OSATS score remained a predictor in the multivariable analysis, whereas the separate components of the uaFRS did not, except for pancreatic texture. This could suggest that surgical skill could add to the uaFRS score. This can help in developing training programs for the RPD through grading the trainees via the, now clinically validated, OSATS score. Also, the found threshold can be used in training programs to set a minimum performance cutoff for the participants (eg, European LEARNBOT program).

Our study has some limitations that should be taken into account. First, we only assessed the modified Blumgart pancreatic anastomosis. This makes generalizability of the present findings for other types of pancreatic anastomoses uncertain. Second, the proposed combined scoring method requires 2 graders, which may limit feasibility and reproducibility in low-resource settings. We are planning to validate grader qualification in a future study (LEARNBOT). Here, we have multiple graders with a different background and experience. The expectation is that with this study we can shed light on how many graders of which background should be used for the OSATS grading. Probably, artificial intelligence features will be able to overcome this issue in coming years. Third, we did not incorporate data on technical specifics, such as type of suturing instrument used, or number of duct-to-mucosa sutures placed relative to the diameter of the pancreatic duct. All participants performed training on a standardized artificial organ model with a 5 mm diameter, only. Fourth, the performance could be impacted by elements of the uaFRS, as smaller (<5 mm) ducts are more difficult to anastomose, and were not part of the training. However, there was no significant association between duct size and OSATS scores, which may reflect the inherent advantage of robotic movement scaling for pancreaticojejunostomy suturing. These findings resonate with the findings of the LAELAPS-3D2D and LAEBOT randomized trials, in which robotic PD anastomoses on artificial organs resulted in better OSATS compared with the (3D)laparoscopy groups.^25^ As OSATS does not access detailed technical errors, for example, ratio of sutures per millimeter of ductal diameter, other technical errors could further explain the residual proportion of POPF (16%) in the highest OSATS quartiles. Also, it is true that the learning curve of robotic PD is determined by multiple factors other than the pancreaticojejunostomy. The safety learning curve for robotic PD in the LAELAPS-3^26^ cohort was 15 procedures whereas the learning curve for operative time for the pancreaticojejunostomy was 34, which reflects the high complexity of the pancreaticojejunostomy compared with the rest of the PD procedures.

In conclusion, the modified OSATS score can identify a learning curve of pancreaticojejunostomy during RPD and may predict the risk of POPF. A higher OSATS score (>49) was associated with significantly lower rate of POPF. Further studies should focus in finding intraoperative and technical variables that may further predict POPF and improve surgical skill and assess the possibility of artificial intelligence-based systems to automatically calculate OSATS scores.

## Supplementary Material

SUPPLEMENTARY MATERIAL
