# Supplementary figures and images for: Video Grading of Pancreatic Anastomoses During Robotic Pancreatoduodenectomy to Assess Both Learning Curve and the Risk of Pancreatic Fistula: A Post Hoc Analysis of the LAELAPS-3 Training Program
Source: Ann Surg. 2023 Jan 20;278(5):e1048–54. doi: 10.1097/SLA.0000000000005796 (PMC10549894; doi:10.1097/SLA.0000000000005796)

## Supplemental digital content 1


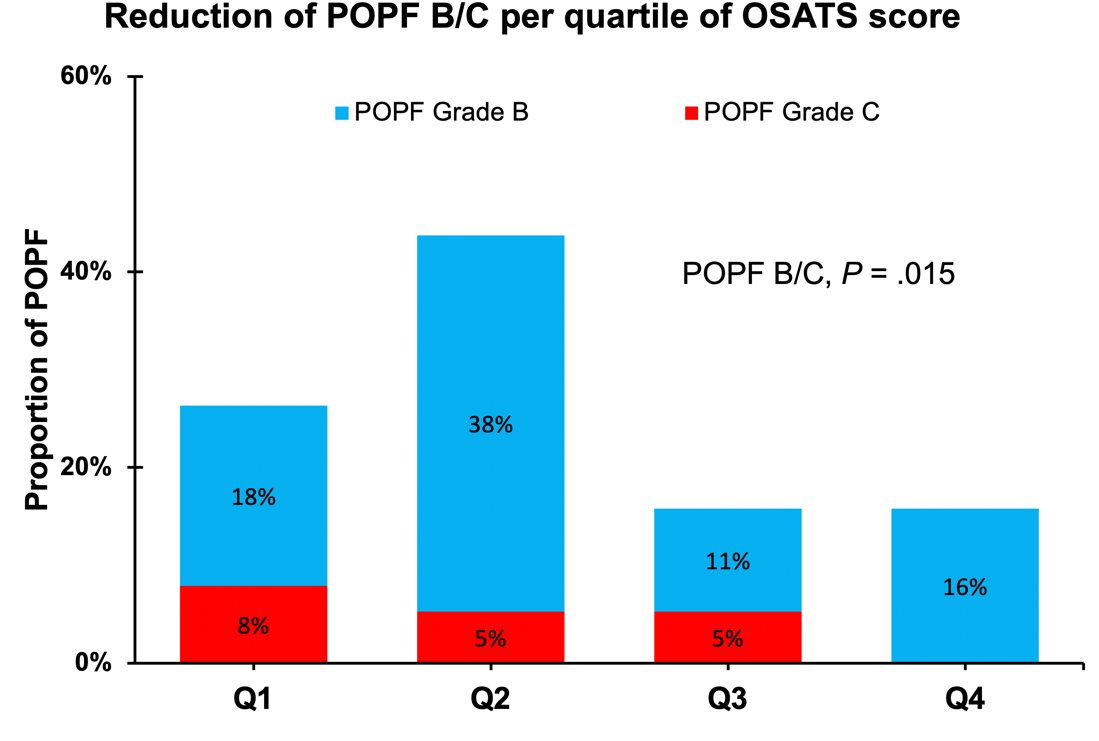


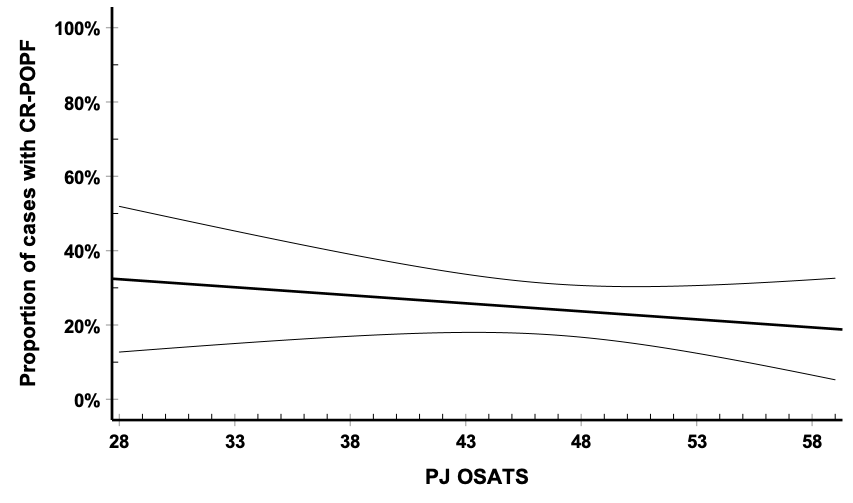


**Linear Reduction of POPF B/C per OSATS**

Supplement: SUPPLEMENTARY MATERIAL [file sla-278-e1048-s001.docx]
