# Supplementary material for: Video Grading of Pancreatic Anastomoses During Robotic Pancreatoduodenectomy to Assess Both Learning Curve and the Risk of Pancreatic Fistula: A Post Hoc Analysis of the LAELAPS-3 Training Program
Source: Ann Surg. 2023 Jan 20;278(5):e1048–54. doi: 10.1097/SLA.0000000000005796 (PMC10549894; doi:10.1097/SLA.0000000000005796)
Supplement: SUPPLEMENTARY MATERIAL [file sla-278-e1048-s002.docx]

## Supplemental digital content 2

| **SDC2. sub analysis Predictive Value of OSATS without neoadjuvant chemo(radio)therapy** | | | | | | | | | | |  |  |
| --- | --- | --- | --- | --- | --- | --- | --- | --- | --- | --- | --- | --- |
|  |  | | **Univariable** | |  |  | | **Multivariable** | |  | |  |
| **Characteristic** | | **Odds Ratio (95%CI)** | | ***P* Value** | | | **Odds Ratio (95%CI)** | | ***P* Value** | | | |
| Quartiles^$^ | | -0.09 | | 0.023 | | |  | |  | | | |
| Quartile 1* | | 1.50 | | 0.419 | | | 1.71 | | 0.486 | | | |
| Quartile 2* | | **3.90** | | **0.003** | | | **3.69** | | **0.005** | | | |
| Quartile 3 | | **1.241** | | **0.678** | | | **1.11** | | **0.882** | | | |
| Quartile 4 | | **Ref** | |  | | | **ref** | |  | | | |
| uaFRS | |  | |  | | |  | |  | | | |
| Age, year increments^$^ | | **0.01** | | **0.863.** | | | **.983** | | **0.449** | | | |
| BMI, kg/m^2^ increments^$^ | | **-0.09** | | **0.269** | | | **.941** | | **0.231** | | | |
| Sex (male) | | **1.16** | | **0.590** | | | **1.14** | | **0.759** | | | |
| Pancreas texture (soft) | | **2.47** | | **0.009** | | | **0.33** | | **0.051** | | | |
| Duct size mm increments^$^ | | **0.07** | | **0.377** | | | **1.14** | | **0.229** | | | |
| 95%CI = 95% confidence interval; uaFRS = Updated alternative fistula risk score. *Values are relative to quartile 4. ^$^Spearman’s rho | | | | | | | | | | |  |  |

When we excluded the 15 patients who received neoadjuvant chemo(radio)therapy the predictability of the uaFRS decreased with 67%. However, the OSATS score only lost 10% of her predictive value. This could be explained by the lower number of patients included in the analysis. While the 67% loss with the uaFRS cannot be explained by the 15 excluded patients. It is most likely that the patients who underwent chemo(radio)therapy had a hard pancreas. Is it known that these patients have a lower probability for developing POPF. When you exclude these patients from the uaFRS it results in a lower power of predictability of uaFRS. While the OSATS score remained consistent. This suggest that the OSATS score is independent of the uaFRS.
