# Supplementary material for: Video Grading of Pancreatic Anastomoses During Robotic Pancreatoduodenectomy to Assess Both Learning Curve and the Risk of Pancreatic Fistula: A Post Hoc Analysis of the LAELAPS-3 Training Program
Source: Ann Surg. 2023 Jan 20;278(5):e1048–54. doi: 10.1097/SLA.0000000000005796 (PMC10549894; doi:10.1097/SLA.0000000000005796)
Supplement: SUPPLEMENTARY MATERIAL [file sla-278-e1048-s004.docx]

## Supplemental digital content 4

| **SDC4, Table 1. Overview of Studies Investigating a Relation Between OSATS and Postoperative Complications in General Surgery** | | | | | |
| --- | --- | --- | --- | --- | --- |
| **Year** | **Journal** | **Author** | **Title** | **Methods** | **Conclusion** |
| 2010 | Minimally Invasive Therapy & Allied Technologies | Mori^27^ | Skill accreditation system for laparoscopic gastroenterologic surgeons in Japan | Scoring: The Japan Society for Endoscopic Surgery (JSES) Endoscopic Surgical Skill Qualification System, two or three blinded graders.  Procedure(s): 1,114 applying surgeons, all in categories biliary tract, esophagus, stomach, colon, spleen, endocrine.  Main outcome: Complications in subgroups of accreditation by the Japan Society for Endoscopic Surgery (JSES) has established an Endoscopic Surgical Skill Qualification System at 70 points. | Surgeons accredited by this system experienced less frequent complications when compared to those who failed. |
| 2013 | NEJM | Birkmeyer^9^ | Surgical Skill and Complication Rates after Bariatric Surgery | Scoring: OSATS: Various domains of technical skill on a scale of 1 to 5 (with higher scores indicating more advanced skill) by at least 10 blinded peers.  Procedure(s): 10,343 patients, laparoscopic gastric bypass.  Main outcome: Complication- [medical and postoperative] and 30-day mortality rates in quartiles of OSATS scores. | The technical skill of practicing bariatric surgeons varied widely, and greater skill was associated with fewer postoperative complications and lower rates of reoperation, readmission, and visits to the emergency department. |
| 2016 | JAMA Surg. | Scally^21^ | Video Ratings of Surgical Skill and Late Outcomes of Bariatric Surgery | Scoring: OSATS: Various domains of technical skill on a scale of 1 to 5 (with higher scores indicating more advanced skill) by at least 10 blinded graders.  Procedure(s): 3,631 patients, laparoscopic gastric bypass.  Main outcome: Excess body weight loss at one year postoperatively in quartiles of OSATS scores. | In contrast to its effect on early complications, surgical skill did not affect postoperative weight loss or resolution of medical comorbidities at 1 year after laparoscopic gastric bypass. |
| 2016 | Annals of Surg. | Hogg^10^ | Grading of Surgeon Technical Performance Predicts Postoperative Pancreatic Fistula for Pancreaticoduodenectomy Independent of Patient-related Variables | Scoring: (1) Pancreatico-jejunostomy step-by-step variables [PJ-specific variables (PJVs); max = 115]; and (2) the Objective Structured Assessment of Technical Skills (OSATS) score by 2 blinded surgeons.  Procedure(s): Modified Blumgart pancreatico-jejunostomy in pancreatoduodenectomy  Main outcome: POPF was diagnosed using International Study Group definition. | This is the first study to demonstrate that technical scoring of a surgeon's performance independently predicts patient outcomes in pancreatic surgery. |
| 2016 | Surg. Oncol. | Paterson^28^ | Videotaping of Surgical Procedures and Outcomes Following Extraperitoneal Laparoscopic Radical Prostatectomy for Clinically Localized Prostate Cancer | Scoring: Video Recorded Extraperitoneal Laparoscopic Radical Prostatectomy Score by independent peer review surgeons.  Procedure(s): 200 patients, Extraperitoneal Laparoscopic Radical Prostatectomy.  Main outcome: Self-reported surgical complications, re-admissions, functional, and oncological outcomes based on a common identifier called as community health index (CHI) number. | Quality of surgical procedure assessed by independent third party videotaping score predicted early resumption of continence following Extraperitoneal laparoscopic radical prostatectomy, however, it did not predict complications, oncological or functional outcome ... |
| 2016 | Surgery | Varban^19^ | Surgical Skill in Bariatric Surgery: Does Skill in One Procedure Predict Outcomes for Another? | Scoring: OSATS, 10 or more blinded peers.  Procedure(s): 20 surgeons, standard laparoscopic gastric bypass procedure.  Main outcome: Surgeons were divided into quartiles for skill in performing gastric bypass and their outcomes within 30 days after sleeve gastrectomy were compared. Multivariate logistic regression analysis was utilized to adjust for patient risk factors. | Video ratings of surgical skill with laparoscopic gastric bypass do not predict outcomes with laparoscopic sleeve gastrectomy. |
| 2017 | Endourology | Goldenberg^29^ | Surgeon Performance Predicts Early Continence After Robot-Assisted Radical Prostatectomy | Scoring: Global evaluative assessment of robotic skill (GEARS) and the generic error rating tool (GERT), one blinded observer with expertise in intraoperative video analysis.  Procedure(s): 48 patients, Robot-Assisted Radical Prostatectomy.  Main outcome: Continence status at 3 months postoperatively, defined as patient use of less than or equal to a single precautionary pad. | Our study generates the hypothesis that there may be a link between surgeon technical performance and functional outcomes in RARP. |
| 2019 | Annals of Surgery | Fecso^30^ | Technical Performance as a Predictor of Clinical Outcomes in Laparoscopic Gastric Cancer Surgery | Scoring: OSATS and Generic Error Rating Tool instruments,  Procedure(s): 61 patients, laparoscopic gastrectomy for cancer.  Main outcome: Major short-term complications. | Intraoperative technical performance predicts major short-term outcomes in laparoscopic gastrectomy for cancer. |
| 2019 | Annals of Surgery | Varban^31^ | Evaluating the Effect of Surgical Skill on Outcomes for Laparoscopic Sleeve Gastrectomy | Scoring: Modified OSATS.  Procedure(s): 25 surgeons, laparoscopic sleeve gastrectomy.  Main outcome: Risk-adjusted 30-day complication rates, 1-year weight loss among cases between surgeons rated in the top and bottom quartiles according to skill. | Peer ratings for surgical skill varied for laparoscopic sleeve gastrectomy but did not have a significant impact on overall complication rates. |
| 2020 | JAMA Surg. | Curtis^32^ | Association of Surgical Skill Assessment With Clinical Outcomes in Cancer Surgery | Scoring: Blinded objective analysis using a bespoke performance assessment tool developed with a 62-international expert Delphi exercise and workshop, interview, and pilot phases.  Procedure(s): 176 patients with clinical T1 to T3 rectal adenocarcinoma 15 cm or less from the anal verge. Laparoscopic total mesorectal excision by 34 credentialed surgeons.  Main outcome: Histopathological (plane of mesorectal dissection, ALaCaRT composite end point success [mesorectal fascial plane, circumferential margin, ≥1 mm; distal margin, ≥1 mm]) and 30-day morbidity. End points were analyzed using surgeon quartiles defined by tool scores. | Intraoperative surgical skill can be objectively and reliably measured in complex cancer interventions. Substantial variation in technical performance among credentialed surgeons is seen and significantly associated with clinical and pathological outcomes. |
| 2020 | JAMA Surg. | Stulberg^20^ | Association Between Surgeon Technical Skills and Patient Outcomes | Scoring: rated by at least 10 blinded peer surgeons and 2 expert graders.  Procedure(s): 17 practicing surgeons, colorectal and non-colorectal procedures.  Main outcome: Any complication, mortality, unplanned hospital readmission, unplanned reoperation related to principal procedure, surgical site infection, and death or serious morbidity. | The findings of this study suggest that there is wide variation in technical skill among practicing surgeons, accounting for more than 25% of the variation in patient outcomes. Higher colectomy technical skill scores appear to be associated with lower complication rates for colectomy and for all other procedures performed by a surgeon. |
| 2020 | JACS | Varban^22^ | Peer Assessment of Operative Videos with Sleeve Gastrectomy to Determine Optimal Operative Technique | Scoring: Technical quality of 9 operative maneuvers (mobilization of the fundus, stapler location, and sleeve width) by 10 blinded peers. An "optimal sleeve gastrectomy score" (OSGS) was calculated as a percentage of the total possible optimal maneuvers performed.  Procedure(s): Practicing bariatric surgeons (n = 30), typical sleeve gastrectomy.  Main outcome: Risk-adjusted 30-day complication rates and 1-year weight loss in the top and bottom quartile for OSGS. | Sleeve gastrectomy videos thought to have "optimal" technique by peer surgeons were associated with lower complication rates. Understanding how to quantify and assess optimal vs suboptimal techniques can serve as a guide for surgeons to improve their practice. |
